# *Lactobacillus* Is Associated With Disease in Pulmonary Arterial Hypertension: A Prospective Cohort Study

**DOI:** 10.1002/cph4.70161

**Published:** 2026-04-28

**Authors:** Arun Jose, Senu Apewokin, Nicholas J. Ollberding, Qing Duan, Jennifer Trannguyen, Sasha Z. Prisco, Thenappan Thenappan, Anna R. Hemnes, Jean M. Elwing

**Affiliations:** ^1^ Department of Medicine University of Cincinnati Cincinnati Ohio USA; ^2^ Department of Pediatrics Cincinnati Children's Hospital Medical Center Cincinnati Ohio USA; ^3^ Cardiovascular Division, Department of Medicine University of Minnesota Minneapolis Minnesota USA; ^4^ Department of Medicine Vanderbilt University Nashville Tennessee USA

**Keywords:** gastrointestinal microbiome, metabolome, pulmonary arterial hypertension, pulmonary hypertension

## Abstract

**Background:**

Gut dysbiosis and gut‐derived metabolites have been linked to pulmonary arterial hypertension. However, associations between specific microbes, and corresponding metabolites, with pulmonary arterial hypertension disease severity is limited.

**Methods:**

This was a prospective cohort study of patients with pulmonary arterial hypertension undergoing right heart catheterization, with pulmonary artery blood subject to nuclear magnetic resonance metabolomics, and simultaneous stool sample shotgun metagenomics. Validation of metabolite levels with disease severity was done in an independent cohort of pulmonary arterial hypertension patients with blood samples from right heart catheterization testing.

**Results:**

The presence of *Lactobacillus* species in the gut microbiome of pulmonary arterial hypertension patients was associated with less severe pulmonary hemodynamics and echocardiographic right ventricular dysfunction. Higher threonine levels were associated with more favorable pulmonary hemodynamic characteristics in both prospective and independent validation cohorts of pulmonary arterial hypertension patients.

**Conclusions:**

Detectable *Lactobacillus* species in the gut microbiome of pulmonary arterial hypertension patients are associated with more favorable pulmonary hemodynamic and right ventricular characteristics. Circulating gut‐derived metabolites may also be involved. Further investigation into the relationship between gut microbial *Lactobacillus*, circulating metabolites, disease severity, and clinical outcomes in pulmonary arterial hypertension may be warranted.

## Graphical abstract

Study overview: simultaneous right heart catheterization, gut microbiome analysis, and plasma blood metabolomics in a cohort of prevalent pulmonary arterial hypertension patients. Key findings: gut microbial *Lactobacillus* species associated with altered gut‐derived metabolite levels, superior measures of right ventricular structure and function, and lower pulmonary hypertension disease severity.

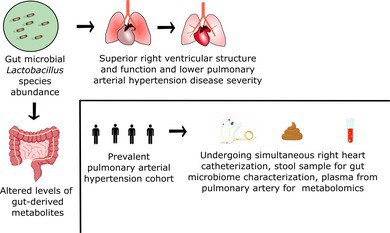

## 1 Introduction

Gut microbial dysbiosis (loss of global diversity, deficiency of symbiotic microbes, overgrowth of pathogenic microbes) has been shown to discriminate patients with pulmonary arterial hypertension (PAH) (Su et al. 2025; Moutsoglou et al. 2022; Kim et al. 2020; Jose et al. 2022) from non‐diseased controls. Modification of the gut microbiome via fecal microbiota transplantation or antibiotic administration, targeting this underlying gut microbial dysbiosis, shows promise in protecting against the development or progression of PAH (Moutsoglou et al. 2025; Chen et al. 2025) in experimental rodent PAH models and small human pilot studies. Multiple gut‐derived metabolites and pathways, including short‐chain fatty acids, choline metabolism, immune dysregulation, and inflammation, are implicated as possible intermediaries regulating this relationship between the gut microbiome and pulmonary vasculature (Moutsoglou et al. 2022, 2025; Chen et al. 2025; Huang et al. 2022). Unfortunately, differences in study design, timing of sample acquisition, and inclusion/exclusion criteria make comparisons across cohorts difficult, and known links between the gut microbiome, circulating metabolome, and PAH disease remain inconsistent. We hypothesized that simultaneous characterization of the gut microbiome, circulating metabolome, and pulmonary hemodynamic measures of PAH disease severity, performed in PAH patients undergoing right heart catheterization (RHC), would validate prior work, address some of these limitations, and identify the most promising links between the gut microbiome, circulating metabolome, and PAH disease for future investigation and development.

## 2 Materials and Methods

This was a prospective cohort study of adult PAH patients between the ages of 18 and 65, screened and enrolled at the University of Cincinnati between October 1, 2022 and December 31, 2024. Eligible participants were diagnosed with PAH by a pulmonary hypertension specialist based on the 7th World Symposium diagnostic criteria for PAH (mean pulmonary artery pressure [mPAP] at rest > 20 mmHg, pulmonary capillary wedge pressure ≤ 15 mmHg, pulmonary vascular resistance [PVR] > 2 Wood Units) (Kovacs et al. 2024), had a body mass index (BMI) < 35 kg/m^2^, and were undergoing right heart catheterization (RHC) testing. Those with comorbid conditions that could affect gut microbial composition (liver cirrhosis, inflammatory bowel disease) (Bajaj et al. 2014; Swirkosz et al. 2023), or those with use of antibiotics, probiotics, or immunosuppressive medications in the three months preceding screening, were excluded. This study was reviewed and approved by our institutional review board (IRB 2019‐0234).

Following consent and enrollment, study participants completed outpatient RHC testing in the standard fashion. No subjects were receiving intravenous fluids at the time of sample collection. Blood samples from the pulmonary artery were obtained for nuclear magnetic resonance (NMR) metabolomics. All samples for metabolomics were tested in a single batch, and a total of 49 metabolites were present in sufficient quantities to be quantified when cross‐referenced with reference spectra from the Human Metabolome Database (Hasson et al. 2022; McCauley et al. 2023). NMR metabolomics testing was untargeted and based on metabolite concentrations in the samples tested; thus, only the most abundant metabolites able to be quantified were reported. A full list of quantified metabolites is available in the online data supplement. Within a week of RHC testing, subjects also provided stool samples for microbiome shotgun metagenomics (Wishart et al. 2007; Chen et al. 2018; Constantinides et al. 2023; Shaw and Yu 2025; Beghini et al. 2021). An abundance threshold of 0.01% was used to determine if a given microbial species was present or absent. When comparing metabolites by gut *Lactobacillus* abundance, those metabolites significantly associated with RHC hemodynamics or composite hemodynamic risk status were also included for comparison.

For the validation cohort, blood plasma samples from PAH patients obtained at the time of RHC were acquired from two additional centers (University of Minnesota and Vanderbilt University). Samples were subject to NMR metabolomics also in a single batch, with only candidate metabolites from the prospective cohort (correlated with measures of PAH hemodynamics, at a significance threshold of 10%) measured in the validation cohort: ascorbate, isopropanol, mannose, N‐N‐dimethyglycine, threonine, ornithine, glutamate, and sn‐glycero‐3‐phosphocholine. Additional clinical metadata (demographics, RHC hemodynamics at time of sample collection) were also obtained.

### 2.1 Statistical Analyses

Data are reported as median (inter‐quartile range) or frequency (percentage) for continuous or categorical variables respectively. Natural log‐transformed metabolite levels were used for analyses. Comparison of sub‐groups on baseline characteristics was performed using Wilcoxon Rank Sum tests or Fisher's Exact tests for continuous or categorical variables respectively. Thermodilution cardiac output (CO) and cardiac index (CI) were preferred for calculations and analyses (Opotowsky et al. 2017) in the prospective cohort. In the validation cohort, only indirect Fick estimates were available. Regression models were adjusted for demographics (age, sex, race), BMI, and institution where sample was collected in the validation cohort.

Spearman correlations were used to evaluate the relationship between estimates of bacterial alpha‐diversity as well as *Lactobacillus* species relative abundance with RHC hemodynamic parameters (McMurdie and Holmes 2013). Ordinations of the first two principal coordinates analysis axes generated from the Bray–Curtis dissimilarity matrix were used to assess whether samples clustered according to RHC hemodynamics. Differentially abundant species, KEGG orthologs, and MetaCyc pathways were identified using MaAsLin2 as implemented in the MaAsLin2 package (version 1.20.0) (Oksanen et al. 2022; Mallick et al. 2020). Associations between pulmonary hemodynamics (mPAP, PVR, CI) and gut bacterial differential abundance was performed using both MaAsLin2 and LinDA, with only species present in at least 20% of all samples included in the analysis, and both an absolute log‐2 fold change of > 0.7 and a false discovery rate *p*‐value of < 0.25 threshold used for reporting of species. Associations between pulmonary hemodynamics and the top metabolic pathways predicted by MetaCyc were based on an absolute log‐2 fold change of 0.7 and a false discovery rate *p*‐value of < 0.25 threshold. Metabolites identified as potentially related to PAH hemodynamic measures of disease severity were subjected to pathway analysis, cross referencing metabolic pathways for generation or utilization with gut microbial metagenomics data using MetaCyc. For threonine this included KEGG pathways K00133 (aspartate semialdehyde dehydrogenase), K00872 (homoserine kinase), K00928 (aspartate kinase), K017233 (threonine synthase), K12524 and K12525 (bifunctional aspartokinase homoserine dehydrogenase). All analyses were performed using the R software environment for statistical computing and graphics version 4.4.0 (R Foundation for Statistical Computing, Vienna, Austria).

## 3 Results

**FIGURE 1:**
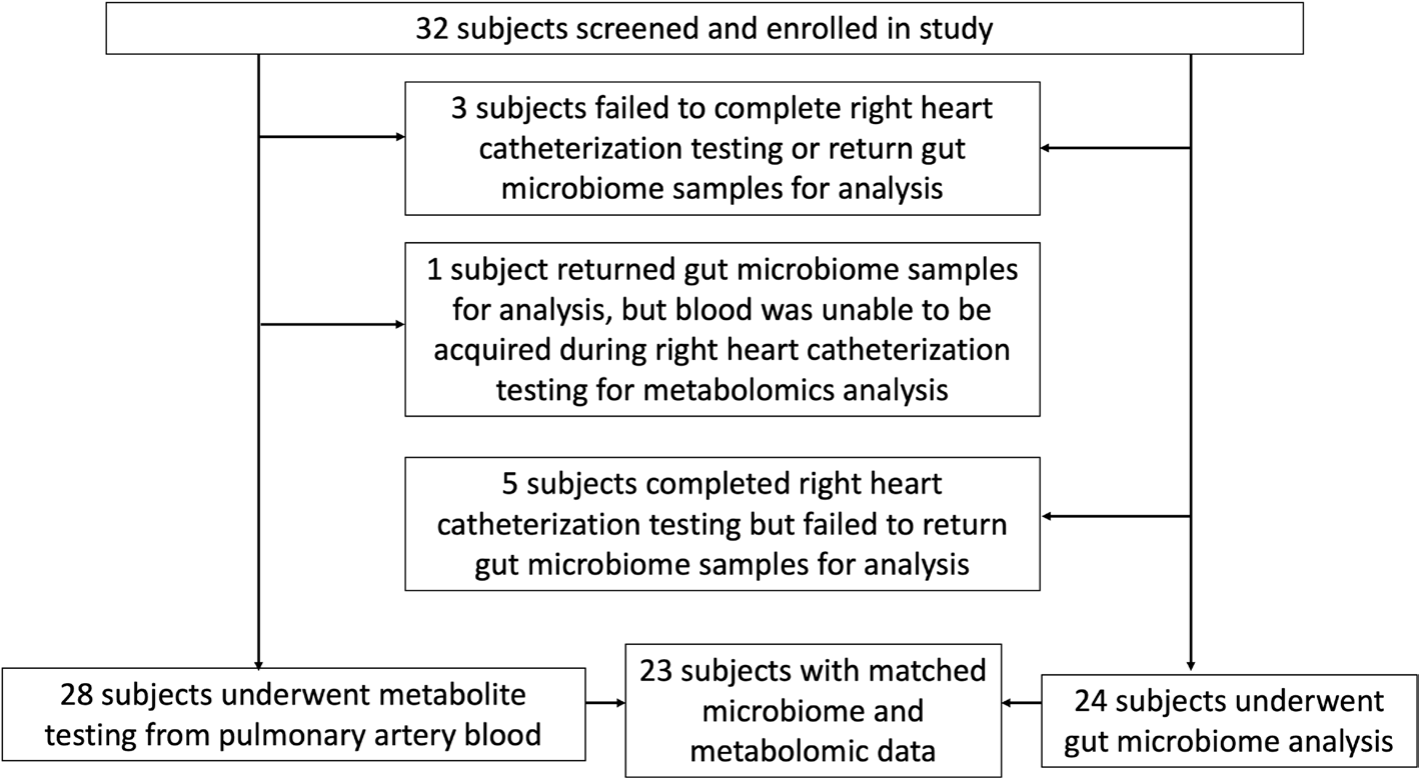
Flow of patients through the study for gut microbiome and metabolome testing during right heart catheterization.

A total of 32 prevalent PAH subjects were screened and enrolled in this study, of which 24 completed RHC and returned fecal samples for analysis, and 23 subjects had simultaneous gut microbiome and circulating metabolite data present for analysis (Figure 1).

The characteristics of patients included in the prospective (University of Cincinnati) cohort analysis for gut microbiome analysis are provided in Table 1. The majority of the cohort was female sex (79%), White race (79%), non‐obese (median BMI 27.2 kg/m^2^), with idiopathic PAH (67%). The majority of the cohort was on either triple oral PAH therapy or parenteral prostacyclin therapy (58%), with a median REVEAL 2.0 risk score of 6 and a median six‐minute walk test distance (6MWT) of 326 m. The median PVR was 4.5 WU (IQR 2.7–6.4) at the time of sample acquisition. Echocardiographic abnormalities were prevalent, with a median tricuspid valve regurgitant jet velocity of 3.2 m/s (IQR 2.9–4.0), a median right ventricular internal diameter in diastole of 3.6 cm (IQR 3.1–3.9), and the presence of subjective dilation or reduced systolic function in 63% of the cohort at the time of sample acquisition.

**TABLE 1:** Baseline characteristics of cohort for microbiome data (*n* = 24).

Variable	Median/F	IQR/%
Age (years)	51	37–63
Gender (female)	19	79%
Race		
White	19	79%
Black	5	21%
Other	1	4%
BMI (kg/m^2^)	27.2	22.9–31.0
Etiology of PAH		
Idiopathic	16	67%
Connective tissue disease associated	6	25%
Heritable	1	4%
Congenital heart disease associated	1	4%
PAH Rx		
None	1	4%
Oral monotherapy	4	17%
Dual oral therapy	5	21%
Triple oral or parenteral	14	58%
Diagnostic mPAP (mmHg)	48	40–53
Diagnostic PCWP (mmHg)	10	7–12
Diagnostic CO (L/min)	5.0	3.6–6.0
Diagnostic PVR (WU)	7.8	4.4–12.5
RA pressure (mmHg)	5	4–8
mPAP (mmHg)	33	30–40
PCWP (mmHg)	10	9–14
CO (L/min)	5.5	4.6–7.0
CI (L/min/m^2^)	4.1	2.5–6.3
PVR (WU)	4.5	2.7–6.4
Left ventricular ejection fraction (%)	55	54–55
TAPSE (cm)	2.1	1.8–2.3
TRV (m/s)	3.2	2.9–4.0
RVID diastole (cm)	3.6	3.1–3.9
RV dilation (subjective)	12	50%
RV reduced function (subjective)	11	46%
RV either dilation or reduced function (subjective)	15	63%
FEV1 (%)	81	59–92
FVC (%)	79	72–97
FEV1/FVC (%)	74	70–78
TLC (%)	92	77–101
DLCO (%)	71	49–78
REVEAL risk score at RHC	6	4–8
6MWT (m)	326	256–380

Abbreviations: 6MWT, six‐minute walk test distance; BMI, body mass index; CI, cardiac index; CO, cardiac output; mPAP, mean pulmonary artery pressure; PCWP, pulmonary capillary wedge pressure; PVR, pulmonary vascular resistance; RA, right atrial pressure; RVID, right ventricular internal diameter; TAPSE, tricuspid annular plane systolic excursion on transthoracic echocardiography; TRV, tricuspid valve regurgitant jet velocity; WU, Wood Units.

A total of 38 PAH subjects with blood samples acquired at the time of RHC were included in the metabolomics validation cohort (Table 2). This cohort was predominantly female sex (61%) and White race (74%), without obesity (median BMI 28.1 kg/m^2^). Samples came from one of two external sites (76% from University of Minnesota, 24% From Vanderbilt University). Connective tissue disease associated PAH (42%) or idiopathic PAH (34%) were the most common etiologies of PAH present. The median PVR was 6.1 WU (IQR 2.4–6.1 WU), and most patients were on oral monotherapy (39%) or dual oral therapy (43%) for PAH.

**TABLE 2:** Baseline characteristics for metabolomic data validation cohort (*n* = 38).

Variable	Median/F	IQR/%
Age (years)	57	45–64
Gender (female)	23	61%
Race		
White	28	74%
Black	6	16%
Other	4	10%
BMI (kg/m^2^)	28.1	25.1–32.8
Site		
Vanderbilt	9	24%
Minnesota	29	76%
Etiology of PAH		
CTD‐PAH	16	42%
IPAH	13	34%
Other	9	24%
PAH Rx		
None	2	5%
Oral monotherapy	15	39%
Dual oral therapy	13	43%
Triple oral or parenteral	8	21%
RA pressure (mmHg)	6	4–10
mPAP (mmHg)	43	36–50
PCWP (mmHg)	11	10–15
CO fick (L/min)	5.2	3.7–5.9
CI fick (L/min/m^2^)	2.7	2.1–3.1
PVR fick (WU)	6.3	4.6–8.4

Abbreviations: BMI, body mass index; CI, cardiac index; CO, cardiac output; CTD, connective tissue disease associated; IPAH, idiopathic PAH; mPAP, mean pulmonary artery pressure; PAH, pulmonary arterial hypertension; PCWP, pulmonary capillary wedge pressure; PVR, pulmonary vascular resistance; RA, right atrial pressure; WU, Wood Units.

### 3.1 Gut Microbiome

There was no correlation between alpha diversity (a measure of microbial community “richness,” quantified either by observed species or Shannon diversity) and either mPAP or PVR in this cohort (Figure S1, Table S1). An inverse correlation between alpha diversity and thermodilution CI was present (observed species rho = −0.48, *p* = 0.019; Shannon rho = −0.43, *p* = 0.035). Similarly, while there was no correlation observed between measures of beta diversity (a measure of microbial community “difference” between samples, both Bray–Curtis and Jaccard dissimilarity) and either mPAP or PVR, a significant positive association was seen between beta diversity and thermodilution CI (Figures S2 and S3, Table S1).

**FIGURE 2:**
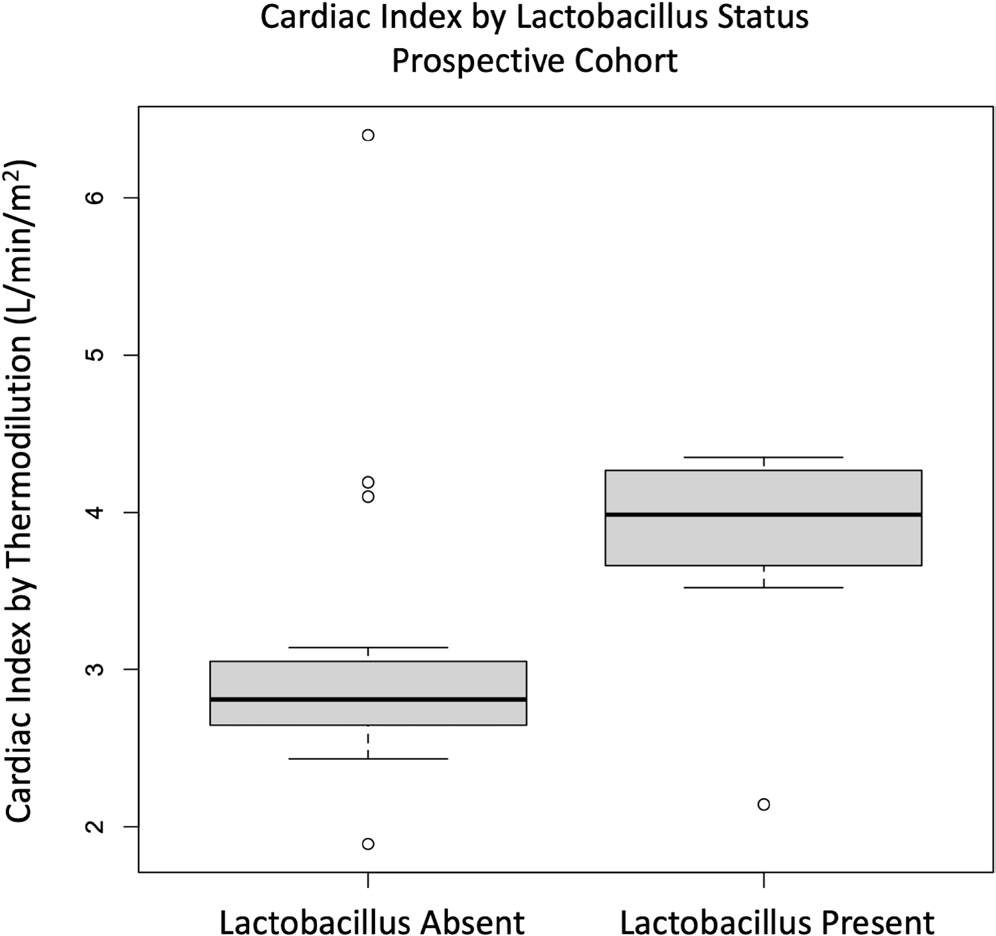
Box plot for differences in cardiac index (L/min/m^2^) on right heart catheterization in prospective cohort between those with gut microbial *Lactobacillus* species present or absent (*p* = 0.036).

Detectable *Lactobacillus* species were present in the minority of the cohort gut microbiome (*n* = 9, 38%). When examining overall differential species abundance for individual species, only *Ventricola* species were associated with pulmonary hemodynamics (PVR) across both MaAsLin2 and LinDA (Table S2). A significant correlation was observed between the presence of any *Lactobacillus* species and both lower PVR (rho = −0.47, *p* = 0.020) and higher CI (rho = 0.41, *p* = 0.044) (Table 3). When compared to those lacking gut *Lactobacillus* species, those with gut *Lactobacillus* species present also had significantly less right ventricular dilation or reduced function on echocardiography (33% vs. 80%, *p* = 0.036), and significantly higher CI (*p* = 0.047) (Figure 2, Table 3).

**TABLE 3:** Gut microbial *Lactobacillus* species and pulmonary hypertension characteristics.

Hemodynamic measure	Rho	*p*
mPAP (mmHg)	−0.25	0.244
TD PVR (WU)	−0.47	0.020
TD CI (L/min/m^2^)	0.41	0.044

Disease measure	*Lactobacillus* present		*Lactobacillus* absent		*p*
	Median	IQR	Median	IQR	
mPAP (mmHg)	33	26–39	33	31–47	0.370
TD PVR (WU)	2.8	1.8–4.6	5.2	3.5–6.4	0.115
TD CI (L/min/m^2^)	4.0	3.7–4.2	2.8	2.6–3.1	0.047
RVID (cm)	3.1	2.9–3.3	3.7	3.6–4.0	0.090
TAPSE (cm)	2.1	1.8–2.5	2.1	1.8–2.3	0.750
RV dilation or reduced function (subjective)	3	33%	12	80%	0.036
REVEAL 2.0 risk score	6	4–9	6	3–8	0.418
6MWT (m)	277	213–349	379	271–440	0.068
Composite hemodynamic “high‐risk” status	8	53%	2	22%	0.210

*Note:* Correlation between hemodynamic measures and *Lactobacillus* abundance via Spearman correlation coefficient. Differences between disease measures and *Lactobacillus* abundance via Wilcoxon Rank Sum test or Fisher's Exact test for continuous or categorical variables respectively.

Abbreviations: 6MWT, six‐minute walk test distance; BMI, body mass index; CI, cardiac index; mPAP, mean pulmonary artery pressure; PVR, pulmonary vascular resistance; RVID, right ventricular internal diameter; TAPSE, tricuspid annular plane systolic excursion on transthoracic echocardiography; TD, thermodilution; TRV, tricuspid valve regurgitant jet velocity; WU, Wood Units.

### 3.2 Circulating Metabolites

**FIGURE 3:**
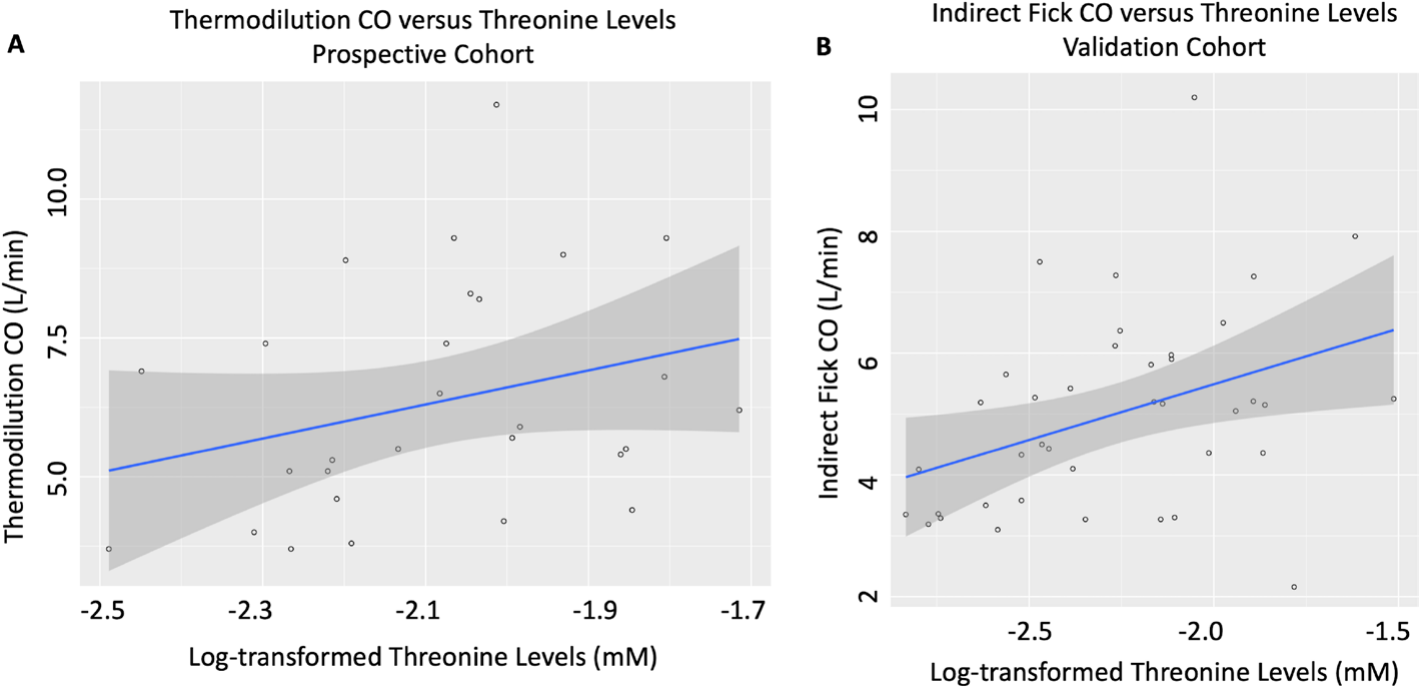
Scatter plot with trendline for relationship between thermodilution cardiac output versus log‐transformed threonine levels in prospective cohort (A) and indirect Fick cardiac output versus log‐transformed threonine levels in validation cohort (B).

In the prospective (University of Cincinnati) cohort, there was a significant correlation between mannose levels and PVR, and between threonine levels and PVR (Table S3). Examining these metabolites in the validation cohort (Table S4), only a significant relationship between threonine and CO was observed after multivariable adjustment (Estimate 1.92, Standard Error 0.93, *p* = 0.048) (Figure 3), with a relationship between threonine levels and PVR no longer statistically significant (Estimate −4.1, Standard Error 2.28, *p* = 0.085).

When comparing metabolite levels by gut *Lactobacillus* status, in the 23 subjects with simultaneous gut microbial and circulating metabolite data, we observed a significantly higher 2‐Hydroxyisovalerate and significantly lower Ascorbate, Glutamate, Trimethylamine‐N‐oxide (TMAO), and Uridine levels in those with gut *Lactobacillus* present as compared to those without (Table S5). Other metabolites of interest, such as those identified from the discovery metabolomics analyses (threonine), were not significantly different when stratified by gut *Lactobacillus* abundance.

### 3.3 Metabolic Pathway Analysis

There were no statistically significant associations between the top MetaCyC metabolic pathways predicted by gut microbiome metagenomics and pulmonary hemodynamics (Figure S4). We also found no association between circulating threonine levels and select genes implicated in gut microbiome threonine metabolism (threonine synthase, homoserine kinase, bifunctional aspartokinase/homoserine dehydrogenase, aspartate kinase, aspartate semialdehyde dehydrogenase) (Figure S5).

### 3.4 Clinical Trajectory

This was a very well controlled PAH patient population, no mortality events were recorded from date of sample collection to a censor date of 1/1/2025 (a total of 620 months of follow‐up, or a median of 26 months per subject [inter‐quartile range 20–33 months]). There were three PAH‐related hospitalizations (two for right heart failure with volume overload, one for worsening of PAH) in the gut *Lactobacillus* absent group, versus one hospitalization (for viral pneumonia) in the gut *Lactobacillus* present group (21% vs. 10%). There were three subjects who needed an escalation in their PAH therapeutic regimen in both groups (21% vs. 30%), and two subjects (both in the gut *Lactobacillus* present group) who improved sufficiently to transition from parenteral prostacyclin therapy to a triple oral PAH treatment regimen.

## 4 Discussion

In this prospective cohort study of PAH patients undergoing simultaneous RHC testing, gut microbiome analysis, and circulating plasma metabolomics, we observed that *Lactobacillus* species were deficient from most PAH gut microbiome samples, but that those with detectable gut microbial *Lactobacillus* species had less severe pulmonary hemodynamics and less right ventricular dysfunction on echocardiography. We did not observe consistent associations between circulating metabolites, global gut microbial characteristics, and PAH disease severity in this cohort. Taken together, these results highlight the challenges in studying the so‐called “gut‐lung” axis in PAH, suggest a possible relationship between gut microbial *Lactobacillus* and disease characteristics in PAH, and support further work clarifying the relationship between the gut microbiome, circulating metabolome, and PAH disease.

Although in the minority, PAH patients with detectable *Lactobacillus* species in their gut microbiome had significantly lower PVR and higher CI on RHC testing. They also demonstrated less subjective right ventricular dilation and dysfunction on echocardiography obtained at the time of sample collection. All hospitalizations for PAH complications occurred in those without gut *Lactobacillus* present, and all treatment de‐escalations from parenteral prostacyclin occurred in those with gut *Lactobacillus* present. Our results appear to support prior work linking gut *Lactobacillus* species to PAH disease severity. Prior epidemiologic studies suggest gut microbial *Lactobacillus* species are detectable in the minority of healthy North American adults, irrespective of PAH status (Ghosh et al. 2020; Prisco et al. 2025), similar to the minority observed in this study. In PAH patients specifically, however, *Lactobacillus* deficiency has previously been associated with worse right ventricular function (fractional area change on echocardiography), and *Lactobacillus* supplementation has been shown to improve both right ventricular structure and function in rodent monocrotaline PAH models (Prisco et al. 2025; Dong et al. 2023). Although a definitive link between gut microbial *Lactobacillus* enrichment, circulating metabolome, and PAH right ventricular dysfunction was not observed in our study, we did find those with gut microbial *Lactobacillus* species demonstrated significantly lower circulating levels of the gut‐derived metabolite TMAO. This is intriguing and noteworthy because in cardiovascular disease patients TMAO is known to be an independent risk factor for both cardiovascular events (myocardial infarction, heart failure) and mortality (Moludi et al. 2021; Ding et al. 2025; Wang et al. 2015), and *Lactobacillus* supplementation significantly reduces circulating TMAO levels in these patients (Spasova et al. 2024). Additionally, higher circulating levels of TMAO have been observed in PAH patients with more severe disease (Moutsoglou et al. 2022; Huang et al. 2022; Yang et al. 2022), and suppression of gut microbial TMAO production has been shown to alleviate right ventricular dysfunction in both monocrotaline and hypoxia rodent PAH experimental models (Huang et al. 2022). Taken together, our data reinforce the link between gut microbial *Lactobacillus* and prognostic disease characteristics in PAH, while suggesting the gut‐derived metabolite TMAO may be a mediator of this relationship. Further studies examining the effects of gut microbial *Lactobacillus* manipulation to circulating TMAO levels and right ventricular morphology in PAH patients, or manipulation of TMAO levels for therapeutic benefit in PAH, will help to test these hypotheses and clarify the link between gut microbial *Lactobacillus*, gut‐derived TMAO, and PAH disease severity and outcomes.

We failed to observe a consistent association between the gut microbiome, circulating metabolome, and PAH disease severity in this cohort. Although we did observe a potentially beneficial effect of higher threonine levels on both CO and PVR, the association remained weak after multivariable adjustment. Plasma threonine levels have previously been associated with development of PAH in the literature (Wawrzyniak et al. 2024; Xu et al. 2022), but these associations are similarly weak and speculative. Additionally, in our cohort, there was no corresponding association between plasma threonine levels and gut microbial expression of genes involved in threonine metabolism (which, as an essential amino acid, is dependent upon dietary intake), and threonine levels did not differ by gut *Lactobacillus* abundance. Thus, the significance of threonine as it relates to the links between gut microbiome and PAH disease severity remains unclear. Future mechanistic investigations examining the effects of gut microbial manipulation on circulating metabolites may be necessary to fully link the gut microbiome with circulating metabolome in PAH.

In our data, we observed an inverse association between CI and alpha diversity, and no association with either mPAP or PVR. One explanation might be that this reflects adaptive right ventricular response to elevated pulmonary pressures (manifesting as less dilation and dysfunction at a given pressure) rather than absolute differences in pulmonary vascular load. In our data we also observed an association between gut microbial *Lactobacillus* species and both higher CI and less right ventricular dysfunction, as well as a direct association between beta diversity and higher CI. Taken together, this may suggest that PAH gut microbial communities rich in *Lactobacillus* species (i.e., less alpha diversity, but a difference in beta diversity) have a more adaptive right ventricular response to elevated pressures (i.e., higher CI) irrespective of mPAP values, and not fully reflected by PVR calculations (which are not indexed for body surface area, and also incorporate pulmonary capillary wedge pressure measurements). There also remains considerable variability in the association between global measures of gut microbial diversity (alpha and beta diversity) and PAH disease severity on RHC hemodynamics. Some studies have shown more favorable characteristics (higher CO, lower PVR) with higher measures of diversity (Moutsoglou et al. 2022; Kim et al. 2020), whereas others showed no difference or even an inverse relationship (Jose et al. 2022; Chen et al. 2025; Dong et al. 2023). While significant manipulation of the gut microbiome via antibiotics in rodent PAH models ameliorated PAH disease severity and altered global measures of gut microbial diversity (Sanada et al. 2020), fecal microbiota transplantation amongst humans with/without PAH, or from humans to rats with monocrotaline‐induced PAH, or between monocrotaline‐induced rats with/without PAH, failed to significantly affect measures of gut microbial diversity (alpha and beta diversity) (Moutsoglou et al. 2025; Chen et al. 2025). This was despite potential evidence of effectiveness, with fecal microbiota transplantation demonstrating transient changes to gut‐derived metabolites and genes encoding specific metabolic pathway enzymes in human PAH patients, and improved measures of PAH disease and right ventricular function in rodents. While diminished microbial diversity may be a hallmark of PAH disease with some discriminatory utility, given the functional redundancy of the gut microbiome and the complex interactions between species that determine gut‐derived metabolism, it also possible that simplistic measures of diversity (alpha and beta) fail to capture the complexity of the gut microbiome as it relates to PAH disease severity (as seen in our data for example, with no significant correlation between RHC measures of disease severity and either alpha or beta diversity) (Coyte et al. 2015). Focusing on specific gut microbial species (such as *Lactobacillus*) that are consistently linked to disease severity, rather than global measures of diversity, may be more informative in understanding the so‐called gut‐lung axis in PAH disease.

Our study has several strengths, including simultaneous RHC testing with gut microbial and circulating metabolite quantification, a comprehensive list of exclusionary criteria to limit the effect of confounders (advanced age, comorbid medical conditions, significant obesity, the effects of antibiotics, probiotics, and immunosuppressive agents), and the use of both a discovery and independent validation cohort for metabolite analyses. Limitations include a small cohort size which limits statistical power for some analyses, further limited by the fact that not all subjects completed all aspects of the study protocol and had simultaneous gut microbiome and circulating metabolite levels available for analysis, making associations linking specific gut microbial characteristics (i.e., *Lactobacillus* species) to circulating metabolites and RHC hemodynamics more challenging. When comparing those who completed the full protocol (*n* = 23) with those who had missing microbiome or metabolome data due to failure to collect or return samples (*n* = 9), there were no differences between groups except the patient population that was analyzed had significantly higher PVR values (median thermodilution PVR 4.5 WU [IQR 2.7, 6.4 WU] vs. 2.2 WU [IQR 1.3, 3.2 WU], *p* = 0.033). We cannot exclude the possibility this difference (“sicker” patients were included in the study, less‐sick patients did not return samples to complete the study) may have resulted in some selection bias impacting the generalizability of our results. Discovery‐based approaches were used for microbiome and metabolome characterization in the discovery cohort, but correction for multiple testing was only utilized when analyzing the microbiome data, and thus an inflated risk of Type 1 error exists. Differences existed between results of mixed‐effects linear models used to analyze gut microbial differential abundance (MaAsLin2, LinDA), and metabolic pathway analyses were negative, suggesting that the links between functional gut‐associated microbiome signatures and hemodynamic variation remain uncertain in this dataset. Consequently, our work should be viewed as primarily hypothesis generating rather than confirmatory. This study was not designed to assess mechanism, and correlations and associations are not necessarily causative. It is unclear if gut microbial *Lactobacillus* species exert a beneficial effect on PAH disease (“causative”), or are just a consequence/marker of PAH disease burden (“effect”). Future mechanistic studies testing manipulation of gut *Lactobacillus* will be necessary to determine causality. We controlled for multiple potential confounders, but did not control for environmental effects such as diet, exercise, environment, PAH etiology, and PAH targeted therapy, all of which may have confounded the relationship between the gut microbiome and circulating metabolome. NMR was used for metabolite quantification for its high reproducibility and absolute quantification, but different techniques (such as liquid chromatography with tandem mass spectrometry), which are able to quantify a larger number and different set of metabolites, may identify alternative candidate gut‐derived metabolites for further investigation. Thermodilution values were used preferentially when available (in the discovery cohort), but were unavailable in the validation cohort, and the differences between Thermodilution and indirect Fick measurements may have affected our results. This was a heavily treated prevalent PAH population, and findings may not be generalizable to incident PAH patients or those with more severe disease.

## 5 Conclusion

In this study, our results suggest a possible link between gut microbial *Lactobacillus* abundance, PAH disease severity, and clinical outcomes. Additional work is needed to better understand the potential implications of gut microbial *Lactobacillus* in predicting PAH clinical outcomes, and further experimental studies are necessary to test the hypothesis that gut *Lactobacillus* manipulation affects the circulating metabolome and/or has therapeutic benefits in PAH.

## Author Contributions

Conceptualization: A.J., J.M.E. Data acquisition: A.J., S.A., J.T., S.Z.P., T.T., A.R.H., J.M.E. Data analysis: A.J., N.J.O., Q.D. Writing (original draft): A.J. Review/editing: all authors.

## Funding

NIH K23 grant HL16497 (A.J.), 2022 Team PHenomenal Hope research award (A.J.), NIH K08 grant HL168166 (S.Z.P.), American Heart Association Career Development Award 23CDA1049093 (S.Z.P.).

## Disclosure

A.J. reports serving on the consultant and advisory board of Janssen, Merck, and Gossamer Bio, and has received research funding from United Therapeutics in the form of an investigator initiated grant. S.Z.P. is on the speaker's bureau for Merck. T.T. has served on the consultant and advisory board of Actelion, United Therapeutics, Altavant, Aria CV, Gossamer Bio, and Acceleron. A.R.H. has served on the consultant and advisory board of Gossamer Bio, Janssen, Merck, United Therapeutics, and Tenax Therapeutics. J.M.E. has received research grant support from Janssen, United Therapeutics, Liquidia, Gossamer Bio, Bayer, Merck, Altavant, Aerovate, Pulmovant, and serves on the consultant or advisory board of United Therapeutics, Altavant, Aerovate, Pulmovant, Bayer, Gossamer Bio, Liquidia, Merck, and Janssen. S.A., N.J.O., Q.D., and J.T. all have nothing to disclose.

## Ethics Statement

This study was reviewed and approved by the University of Cincinnati institutional review board (IRB 2019‐0234). All subjects gave informed consent prior to study inclusion, and study conduct conformed to recognized standards.

## Conflicts of Interest

The authors declare no conflicts of interest.

## Supporting information


**Data S1:** cph470161‐sup‐0001‐Supinfo1.csv.

**Figures S1–S7:** cph470161‐sup‐0002‐Supinfo2.docx.
**Tables S1–S5:** cph470161‐sup‐0002‐Supinfo2.docx.

## Data Availability

Gut microbiome shotgun metagenomic data generated by this study are deposited and accessible in the NCBI Sequence Read Archive (SUB14731817) on January 11, 2026 or upon publication, whichever comes first. Additional data supporting the findings of this study are available from the corresponding author (A.J.) upon reasonable request. Additional detail on methods is provided in an Supporting Information.
