# Supplementary material for: Lactobacillus Is Associated With Disease in Pulmonary Arterial Hypertension: A Prospective Cohort Study
Source: Compr Physiol. 2026 Apr 28;16:e70161. doi: 10.1002/cph4.70161 (PMC13124308; doi:10.1002/cph4.70161)
Supplement: Supplementary file 2 — Figures S1–S7: cph470161‐sup‐0002‐Supinfo2.docx. Tables S1–S5: cph470161‐sup‐0002‐Supinfo2.docx. [file CPH4-16-e70161-s002.docx]

***Lactobacillus* is associated with disease in pulmonary arterial hypertension: A prospective cohort study**

Arun Jose, Senu Apewokin, Nicholas J. Ollberding, Qing Duan, Jennifer Trannguyen, Sasha Z. Prisco, Thenappan Thenappan, Anna R. Hemnes, Jean M. Elwing

**ONLINE DATA SUPPLEMENT**

Table of Contents

Supplementary Methods………………………………………………………………….Pages 3-7

Supplementary References……………………………………………………………….Pages 8-9

Figure S1……………………...…………………………………………………...……….Page 10

Figure S2……………………...…………………………………………………...……….Page 11

Figure S3……………………...…………………………………………………...……….Page 12

Figure S4……………………...…………………………………………………...……….Page 13

Figure S5……………………...…………………………………………………...……….Page 14

Figure S6...…………………...……………………………………….…………...……….Page 15

Figure S7……………………...……………………………………….…………...………Page 16

Table S1……………………...……………………………………….…………...……….Page 17

Table S2……………………...……………………………………….…………...……….Page 18

Table S3……………………...……………………………………….…………...……….Page 19

Table S4……………………...……………………………………….…………...……….Page 20

Table S5……………………...……………………………………….…………...……….Page 21

Supplementary Methods

Prospective Cohort

Subjects were required to be clinically stable, without changes in their PAH medication regimen for at least 3 months prior to enrollment. Those with underlying diagnoses of short bowel syndrome, chronic thromboembolic pulmonary hypertension, human immunodeficiency virus or common variable immunodeficiency, inflammatory bowel disease (Crohn's or Ulcerative Colitis), or significant liver disease with or without cirrhosis, were excluded. Those with use of antibiotics, probiotic supplements, or use of immunosuppressive medications (including but not limited to: corticosteroids, methotrexate, tacrolimus, mycophenolate mofetil, or similar compounds), in the preceding three months were also excluded. Pregnant or nursing women were excluded. This study was reviewed and approved by our institutional review board (IRB 2019-0234).

Following consent and enrollment, study participants completed RHC testing in the standard fashion. Blood samples were obtained from the pulmonary artery for metabolomics testing, collected in 10cc lithium heparin plasma tubes. Clinical metadata (demographics, past medical history, PAH treatment history, six-minute walk testing results, right heart catheterization hemodynamics, echocardiographic data, pulmonary function testing data, etc.) were obtained from the medical record. The REVEAL 2.0 multicomponent risk score was calculated at the time of study enrollment^1^. Subjects provided a stool sample for gut microbiome analysis within one week of RHC testing.

Validation Cohort

Included patients with PAH had blood samples obtained during RHC testing in lithium heparin or citrated plasma tubes. University of Minnesota samples were obtained from the SVC/RA port, Vanderbilt University samples were obtained from the pulmonary artery port, during RHC. Individual material transfer agreements and data use agreements with each center were established under the current IRB-approved protocol prior to sharing of any biological samples or clinical data. Etiology of pulmonary hypertension (idiopathic PAH, connective tissue associated PAH, drug/toxin induced PAH, congenital heart disease associated PAH, etc.) was determined by the sharing center's pulmonary hypertension specialist. De-identified clinical metadata was shared, including demographics (age, sex, race), BMI, targeted PAH therapy at time of sample collection, and RHC hemodynamics at time of sample collection (right atrial pressure, mPAP, pulmonary capillary wedge pressure, indirect Fick cardiac output [CO] and cardiac index [CI]). PVR was calculated based on supplied RHC hemodynamic data.

Metabolomic Analysis

Following collection, blood plasma samples were stored at minus 80 degrees Celsius until use. All samples from the full cohort were tested simultaneously (one batch). Nuclear Magnetic Resonance (NMR) metabolite quantification experiments were performed at the Cincinnati Children's Hospital Medical Center Translational Metabolomics Facility (RRID:SCR_022636). Plasma samples were processed according to the previously published protocol^2^. Briefly, plasma samples were filtered through pre-washed 3 kDa spin filters, and the filtrate was mixed with NMR buffer (100mM phosphate buffer in D_2_O, pH 7.3, and 1.0 mM TMSP [3-Trimethylsilyl 2,2,3,3-d_4_ propionate], up to the final volume of 600uL. NMR data collection and processing were performed with a Bruker Avance III HD 600 MHz spectrometer using Topspin 3.6 software (Bruker Analytik, Rheinstetten, Germany) as previously described^3^. Plasma metabolites were assigned based on the chemical shifts on 1D ^1^H-NOESY, 2D-TOCSY and -HSQC NMR experiments with reference spectra found in databases, Human Metabolome Database^4^, and Chenomx® NMR Suite profiling software (Chenomx Inc. version 8.1). A total of 49 metabolites were quantified by Chenomx software based on the internal standard, specifically: 2-hydroxybutyrate, 2-hydroxyisovalerate, 2-oxoisocaproate, 3-hydroxybutyrate, 3-hydroxyisovalerate, 2-methyl-2-oxovalerate, acetate, acetoacetate, acetone, alanine, arabinose, ascorbate, asparagine, choline, citrate, creatine, creatinine, dimethylamine, formate, glucose, glutamate, glutamine, glycine, histidine, isobutyrate, isoleucine, ispropanol, lactate, leucine, lysine, mannose, methanol, methionine, N-N-dimethyglycine, O-acetylcarnitine, ornithine, phenylalanine, proline, pyruvate, serine, threonine, trimethylamine-N-oxide, tyrosine, urea, uridine, valine, myo-inositol, and sn-glycero-3-phosphocholine. Metabolite levels are reported in millimolar unless otherwise specified. Log-transformed metabolite levels were used for analyses.

Gut Microbiome Analysis

For metagenomic sequencing, DNA extraction was performed on stool samples. The samples were collected using a DNA/RNA Shield™ fecal collection tube (Zymo Research, Irvine, CA, USA). Following collection, samples were stored at minus 80 degrees Celsius until processing and use. Microbial DNA was extracted from each sample using the QIAamp PowerFecal Pro isolation Kit (Qiagen, Venlo, Netherlands) in conjunction with a semi-automated QIAcube instrument (Qiagen, Venlo, Netherlands). DNA extracts were quantified using the Qubit 1X dsDNA HS Assay Kit (Invitrogen, Waltham, MA, USA) with a Qubit Fluorometer. After DNA extraction, library preparation for short-read sequencing was performed using the Nextera XT DNA Library Preparation Kit (Illumina, San Diego, CA, USA), and libraries were sequenced on an Illumina NovaSeq6000 sequencer machine (Illumina, San Diego, CA, USA) to a depth of 2.5 Gbp per sample.

Quality control included adapter removal, read filtering, and trimming of low-quality regions using Fastp (version 1.0.1)^5^. Default settings were used other than removing reads <75 bases after trimming. Host contaminant read filtering was performed using Hostile (v2.0.1)^6^ against the T2T-CHM13v2.0 human reference genome and IPD-IMGT/HLA index files with the bowtie2 aligner at the default settings. Sylph (version 0.8.1)^7^ was used to generate taxonomic profiles by comparing the containment average nucleotide identity of k-mer sketches in the pre-built GTDB-R226 -c 200 database to those in each sample and then remapping reads at the default settings. The product of the species relative abundance and read counts after quality control and host contaminant removal was used to obtain pseudo counts. Functional profiling of UniRef90 gene families was performed using HUMAnN3 (version 3.9) at the default settings^8^. UniRef90 gene families were assigned to MetaCyc pathways and KEGG orthologs using the built-in scripts in HUMAnN3. Species detected at less than 1e-4 relative abundance were removed prior to statistical analyses to limit the impact of potential false positive classifications.

Statistical Analyses

Species richness and Shannon diversity were calculated using the estimate_richness function in the phyloseq package (version 1.50.0)^9^ after subsampling to 1e+07 reads per sample. Spearman correlations were used to evaluate the relationship between estimates of bacterial alpha-diversity and RHC hemodynamic parameters (e.g., mPAP, PVR, and CI), as well as for correlating these measures with *Lactobacillus* species relative abundance. Ordinations of the first two principal coordinates analysis (PCoA) axes generated from the Bray-Curtis dissimilarity matrix were used to assess whether samples clustered according to different measures such as RHC hemodynamics. RHC hemodynamic measures were dichotomized at the median value for ordination plots. Permutational multivariate analysis of variance as implemented in the adonis2 function in vegan (version 2.6.10)^10^ was used to formally test for differences in the centroids across clusters. Differentially abundant species, KEGG orthologs, and MetaCyc pathways were identified using MaAsLin2 as implemented in the MaAsLin2 package (version 1.20.0)^11^. Models were run under the default settings except for the minimum prevalence for inclusion set to 0.2. RHC hemodynamic measures were scaled to reflect the log2 fold-change for a one SD increase. Features with a Benjamini-Hochberg false discovery rate p-value <0.25 were flagged as potentially differentially abundant. All analyses were performed using the R software environment for statistical computing and graphics version 4.4.0 (R Foundation for Statistical Computing, Vienna, Austria).

Correlations between circulating metabolites and RHC hemodynamic parameters were assessed using Kendall correlation coefficients and log-transformed metabolite levels. Significant correlations were evaluated in the prospective cohort using multivariable regression models adjusted for age, sex, race, and BMI, and in the validation cohort using multivariable regression models adjusted for age, sex, race, BMI, and sample site. A p-value of 0.05 was deemed significant.

References

1. Benza RL, Gomberg-Maitland M, Elliott CG, Farber HW, Foreman AJ, Frost AE, et al. Predicting survival in patients with pulmonary arterial hypertension: the REVEAL risk score calculator 2.0 and comparison with ESC-ERS-based risk assessment strategies. Chest 2019;156(2):323-337.
2. Hasson DC, Watanabe-Chailland M, Romick-Rosendale L, Koterba A, Miner DS, Lahni P, et al. Choline supplementation attenuates experimental sepsis-associated acute kidney injury. Am J Physiol Renal Physiol 2022;323(3):F255-F271.
3. McCauley HA, Riedman AM, Enriquez JR, Zhang X, Watanabe-Chailland M, Sanchez JG, et al. Enteroendocrine cells protect the stem cell niche by regulating crypt metabolism in response to nutrients. Cell Mol Gastroenterol Hepatol 2023;15(6):1293-1310.
4. Wishart DS, Tzur D, Knox C, Eisner R, Guo AC, Young N, et al. HMDB: the human metabolome database. Nucleic Acids Res 2007;35(Database issue):D521-D526.
5. Chen S, Zhou Y, Chen Y, Gu J. fastp: an ultra-fast all-in-one FASTQ preprocessor. Bioinformatics 2018;34(17):i884-i890.
6. Constantinides B, Hunt M, Crook DW. Hostile: accurate decontamination of microbial host sequences. Bioinformatics 2023;39(12):btad728.
7. Shaw J, Yu YW. Rapid species-level metagenome profiling and containment estimation with sylph. Nat Biotech 2025;43(8):1348-1359.
8. Beghini F, McIver LJ, Blanco-Miguez A, Dubois L, Asnicar F, Maharjan S, et al. Integrating taxonomic, functional, and strain-level profiling of diverse microbial communities with bioBakery 3. Elife 2021;10:e65088.
9. McMurdie PJ, Holmes S. phyloseq: an R package for reproducible interactive analysis and graphics of microbiome census data. PLoS One 2013;8(4):e61217.
10. Oksanen J, Simpson GL, Blanchet FG, Kindt R, Legendre P, Minchin PR, et al. vegan: Community Ecology package. R package version 2.6-4 (2022); <https://CRAN.R-project.org/package=vegan>.
11. Mallick H, Rahnavard A, McIlver LJ. MaAsLin2: multivariable association in population-scale Meta-omics studies. R/Bioconductor package (2020); <http://huttenhower.sph.harvard.edu/maaslin2>.

Supplemental Figures

Figure S1

Relationship between measures of alpha diversity (observed species, top row; Shannon diversity, bottom row) and right heart catheterization hemodynamic measures (mean pulmonary artery pressure, thermodilution pulmonary vascular resistance, thermodilution cardiac index).

Figure S2

Relationship between measures of beta diversity (Bray-Curtis dissimilarity) and right heart catheterization hemodynamic measures (mean pulmonary artery pressure, thermodilution pulmonary vascular resistance, thermodilution cardiac index).

Figure S3

Relationship between measures of beta diversity (Jaccard dissimilarity) and right heart catheterization hemodynamic measures (mean pulmonary artery pressure, thermodilution pulmonary vascular resistance, thermodilution cardiac index).

Figure S4

Plot displaying differential MetaCyc pathway analysis across right heart catheterization hemodynamic measures (mean pulmonary artery pressure, thermodilution pulmonary vascular resistance, thermodilution cardiac index). Bars denote log2-fold-change associated with a one-unit increase in hemodynamic measure or biomarker across pathways with absolute log2-fold-changes ≥ 0.7. Green bars denote pathways with a Benjamini-Hochberg false discovery rate p-value of <0.25.

Figure S5


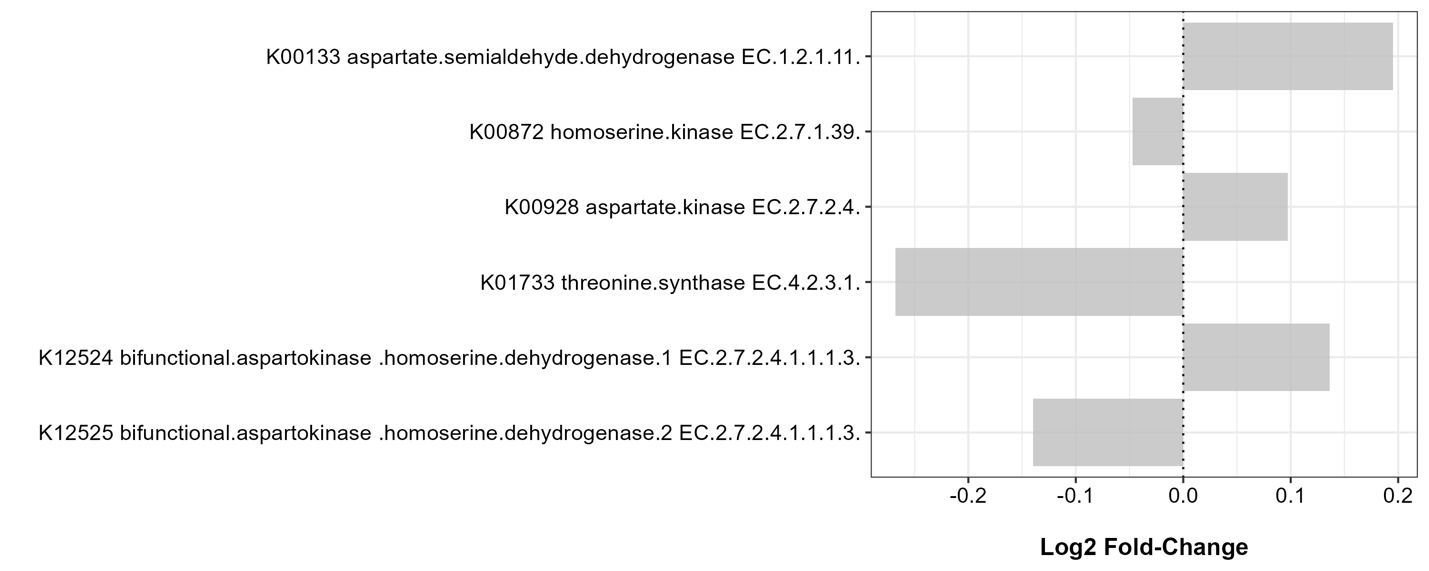


Relationship between circulating plasma threonine levels and gut microbial expression of genes encoding enzymes involved in threonine metabolism.

Figure S6

**
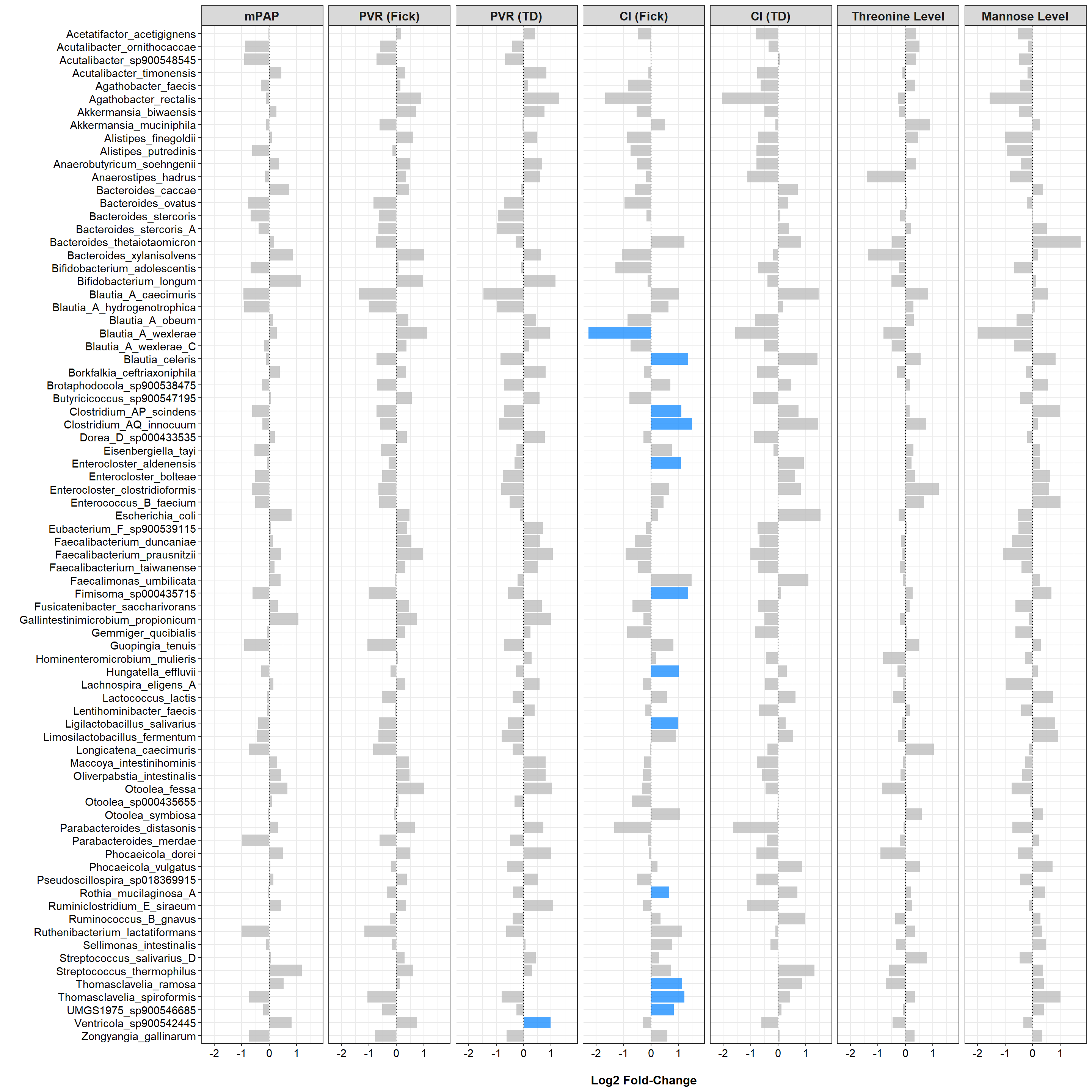
**

Overall differential species abundance analysis versus hemodynamics (mean pulmonary artery pressure, cardiac index, pulmonary vascular resistance) and both Mannose and Threonine levels using MaAsLin2

Figure S7

**
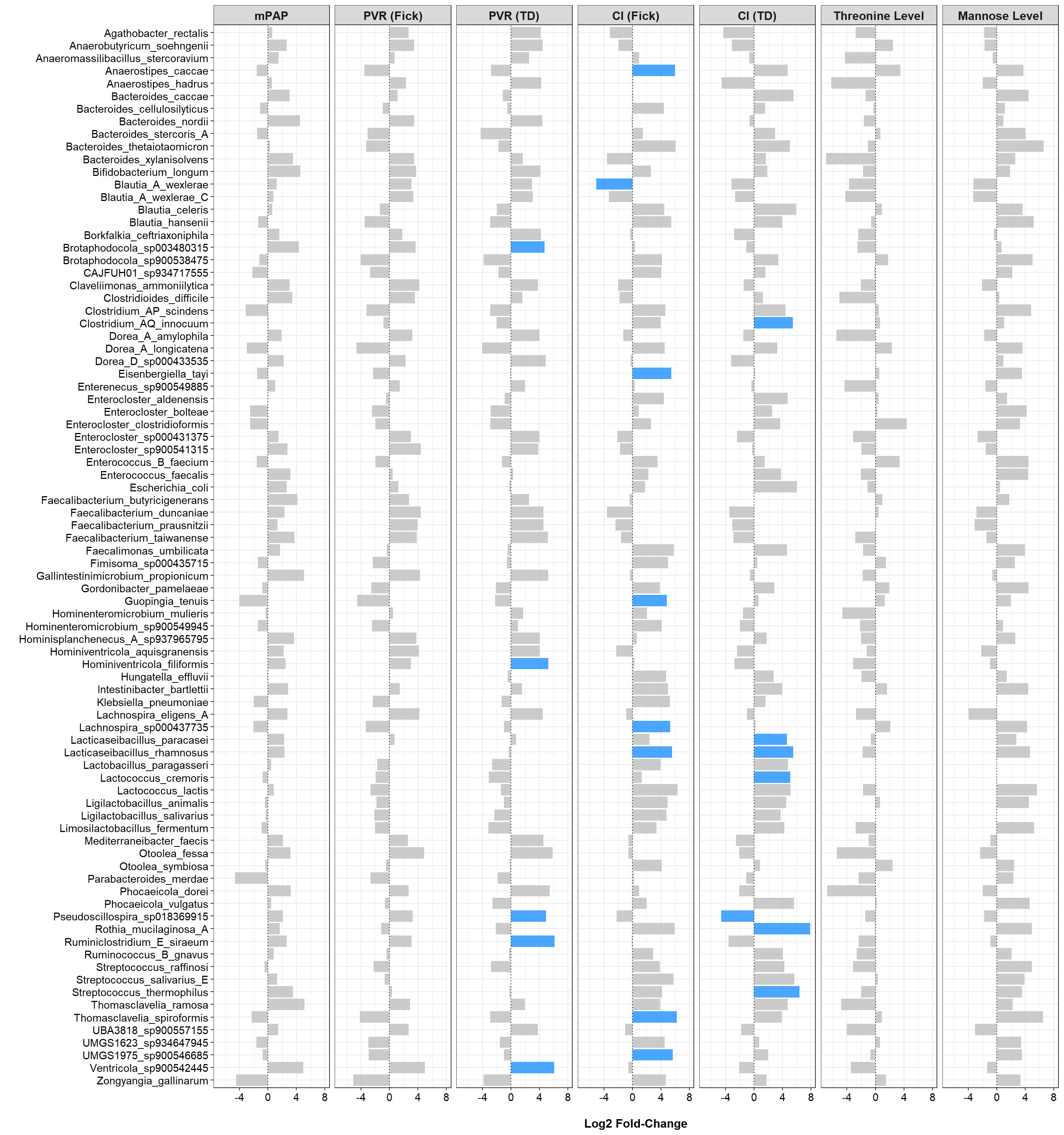
**

Overall differential species abundance analysis versus hemodynamics (mean pulmonary artery pressure, cardiac index, pulmonary vascular resistance) and both Mannose and Threonine levels using LinDA

**Table S1: Gut Microbial Diversity and Pulmonary Hemodynamics**

| **Measure - Alpha Diversity** | **Hemodynamic** | **Rho** | **p-value** |
| --- | --- | --- | --- |
| Observed Species | mPAP | -0.05 | 0.830 |
| Observed Species | TD PVR | 0.20 | 0.360 |
| Observed Species | TD CI | -0.48 | 0.019 |
| Shannon | mPAP | -0.06 | 0.790 |
| Shannon | TD PVR | 0.13 | 0.560 |
| Shannon | TD CI | -0.43 | 0.035 |
|  | | | |
| **Measure - Beta Diversity** | **Hemodynamic** | **R^2^** | **p-value** |
| Bray-Curtis | mPAP | 0.041 | 0.520 |
| Bray-Curtis | TD PVR | 0.053 | 0.144 |
| Bray-Curtis | TD CI | 0.092 | 0.003 |
| Jaccard | mPAP | 0.039 | 0.852 |
| Jaccard | TD PVR | 0.053 | 0.055 |
| Jaccard | TD CI | 0.064 | 0.005 |

Abbreviations: mPAP - mean pulmonary artery pressure; PVR - pulmonary vascular resistance; TD - thermodilution; CI - cardiac index

**Table S2: Gut Microbial Bacterial Species and Pulmonary Hemodynamics**

| **Bacterial Species** | **Hemodynamic Measure** | **Analysis** | **Log2 Fold Change** | **p-value** |
| --- | --- | --- | --- | --- |
| *Ventricola* | TD PVR | MaAsLin2 | 0.98 | 0.030 |
| *Ventricola* | TD PVR | LinDA | 6.08 | 0.147 |

Abbreviations: PVR - pulmonary vascular resistance; TD - thermodilution; CI - cardiac index

**Table S3: Metabolites and PAH Hemodynamics in Prospective Cohort**

| **Kendall Correlation Coefficients for Log-Transformed Metabolite Levels** | | | | |
| --- | --- | --- | --- | --- |
| Metabolite | Correlation mPAP | Correlation PVR | | Correlation CO |
| Mannose | tau = -0.16, p = 0.227 | tau = -0.31, p = 0.020 | | tau = 0.29, p = 0.028 |
| Threonine | tau = -0.16, p = 0.243 | tau = -0.25, p = 0.060 | | tau = 0.27, p = 0.046 |
|  | | | | |
| **Multivariable Linear Regression Models for Metabolite (adjusted for age, sex, race, BMI)** | | | | |
| Log-Transformed Mannose and PVR | | | Estimate: -5.0, STE: 1.98, p=0.020 | |
| Log-Transformed Mannose and CO | | | Estimate: 2.3, STE: 1.29, p=0.088 | |
| Log-Transformed Threonine and CO | | | Estimate: 3.4, STE: 1.70, p=0.061 | |
| Log-Transformed Threonine and PVR | | | Estimate: -6.3, STE: 2.66, p=0.027 | |

Abbreviations: PAH - pulmonary arterial hypertension; mPAP - mean pulmonary artery pressure; PVR - pulmonary vascular resistance; CO - cardiac output; BMI - body mass index

**Table S4: Metabolites and PAH disease severity in Validation Cohort**

| **Kendall Correlation Coefficients for Log-transformed Metabolite Levels** | | | | |
| --- | --- | --- | --- | --- |
| Metabolite | Correlation mPAP | | Correlation PVR | Correlation CO |
| Mannose | tau = 0.21  p = 0.207 | | tau = 0.27  p = 0.103 | tau = -0.07  p = 0.674 |
| Threonine | tau = -0.01  p = 0.967 | | tau = -0.23  p = 0.163 | tau = 0.35  p = 0.030 |
|  | | | | |
| **Multivariable Linear Regression Models (adjusted for age, sex, race, BMI, Site)** | | | | |
| Log-Transformed Mannose and PVR | | Estimate: 3.5, STE: 2.62, p=0.191 | | |
| Log-Transformed Mannose and CO | | Estimate: -0.4, STE: 1.12, p=0.704 | | |
| Log-Transformed Threonine and CO | | Estimate: 1.9, STE: 0.93, p=0.048 | | |
| Log-Transformed Threonine and PVR | | Estimate: -4.1, STE: 2.28, p=0.085 | | |

Abbreviations: PAH - pulmonary arterial hypertension; mPAP - mean pulmonary artery pressure; PVR - pulmonary vascular resistance; CO - cardiac output; BMI - body mass index

**Table S5: Select Metabolites and Gut Microbial *Lactobacillus* Species in Prospective Cohort**

| **Difference in Medians (Wilcoxon Signed Rank Test) for Metabolite Levels** | | | |
| --- | --- | --- | --- |
| Metabolite | Lactobacillus Present  Median (IQR) | Lactobacillus Absent  Median (IQR) | p-value |
| Threonine | 0.133 (0.120, 0.159) | 0.112 (0.107, 0.137) | 0.325 |
| 2-Hydroxyisovalerate | 0.007 (0.006, 0.008) | 0.004 (0.003, 0.006) | 0.045 |
| Ascorbate | 0.013 (0.010, 0.030) | 0.035 (0.022, 0.049) | 0.047 |
| Glutamate | 0.058 (0.049, 0.066) | 0.090 (0.060, 0.108) | 0.045 |
| Trimethylamine-N-Oxide | 0.017 (0.012-0.022) | 0.024 (0.019-0.029) | 0.036 |
| Uridine | 0.003 (0.003, 0.003) | 0.004 (0.003, 0.005) | 0.038 |
